# LRRK2 kinase modulates glucose-stimulated insulin secretion via RAB8 phosphorylation and ciliogenesis

**DOI:** 10.1007/s00018-025-05810-w

**Published:** 2025-07-17

**Authors:** Nevia Dule, Algerta Marku, Alessandra Galli, Francesca Pischedda, Adriano Lama, Michela Castagna, Paola Marciani, Federico Bertuzzi, Giovanni Piccoli, Carla Perego

**Affiliations:** 1 Laboratory of Molecular and Cellular Physiology, Department of Excellence of Pharmacological and Biomolecular Sciences, Università degli Studi di Milano, Via Trentacoste 2, 20134 Milan, Italy; 2 CIBIO, Università Degli Studi di Trento, Dulbecco Telethon Institute, Trento, Italy; 3 Niguarda Cà Granda Hospital, Milan, Italy

**Keywords:** LRRK2; β-cell; RABs, Primary cilium, Insulin, Parkinson disease

## Abstract

Leucine-rich repeat kinase 2 (LRRK2) encodes a multidomain protein whose mutations have been identified as genetic risk factors for Parkinson’s disease (PD), an age-related neurodegenerative disorder. Outside the nervous system, LRRK2 is expressed in multiple tissues, including the endocrine pancreas, but its role here is unknown. Using pharmacological and molecular approaches, we show that LRRK2 kinase activity regulates stimulated insulin secretion by influencing secretory granule trafficking. The PD-associated LRRK2 mutant G2019S, characterized by enhanced kinase activity, increases the basal insulin release in complementary in vitro models and affects the metabolic profile in transgenic mice. Mechanistically, we demonstrate that LRRK2 kinase activity influences the formation of the primary cilium, an antenna-like structure acting as signaling platform to regulate hormones secretion. Specifically, LRRK2 phosphorylates RAB8 in a glucose-dependent manner, facilitating its recruitment to the primary cilium. These findings identify LRRK2 as a regulator of insulin secretion in pancreatic β-cells. Given the role of insulin signaling and glucose homeostasis in the nervous system, our data suggest that LRRK2 may also contribute to PD development through peripheral action.

**Supplementary Information:**

The online version contains supplementary material available at 10.1007/s00018-025-05810-w.

## Introduction

Leucine-Rich Repeat Kinase 2 (LRRK2) is a large protein encompassing several functional domains, including kinase and GTPase enzymatic activities and multiple protein-protein interaction modules [1]. It is expressed in the central nervous system (CNS) and it is among the most important genetic risk factors for Parkinson’s disease (PD) [2], a common, age-dependent, neurodegenerative disorder. PD is characterized by the progressive loss of dopaminergic neurons with subsequent motor alterations such as bradykinesia, rigidity, and resting tremor [3]. Among all the pathological LRRK2 mutations, the substitution G2019S, falling within the kinase domain, is the most frequent one and induces a significant increase in LRRK2 kinase activity both in vitro and in vivo [4–6].

Besides being expressed in the CNS, LRRK2 plays a crucial role also in the periphery [7]. Noteworthy, LRRK2 is expressed in several insulin-sensitive tissues, including muscle and adipose tissue, where it has been implicated in the modulation of the insulin signaling and glucose homeostasis [8–10]. LRRK2 is also expressed in the endocrine pancreas [11], yet its role in this context remains largely unknown. Over the last years, metabolic syndromes such as type 2 diabetes mellitus (T2DM), a chronic disorder characterized by hyperglycaemia and alterations in insulin level and/or insulin action, have been identified as risk factors for PD development [12, 13]. Interestingly, both neurons and pancreatic β-cells are specialized secretory cells that synthesize, accumulate and release neurotransmitters/hormones, a process that requires receptors and transporters to sense different chemical signals as well as a sophisticated secretory machinery including V- and T-SNAREs and RAB proteins, organized in specific microdomains [14–17]. Finally, both cell types have the primary cilium, a microscopic sensory antenna used to gather information about the environment. In the pancreatic β-cells, the cilium is critically involved in regulating β-cell proliferation, glucose sensing and insulin secretion [18–20].

Cumulating evidence links LRRK2 to the endolysosomal system [21, 22]. More recent data have suggested a role for LRRK2 in the formation of the primary cilium, a microtubule-based organelle important for sensing and signaling in eukaryotic cells [23]. LRRK2 executes its many functions via the interaction and phosphorylation of RAB proteins, small GTP-binding proteins involved in the regulation of endomembrane trafficking [6]. In particular, RAB8, RAB10, and RAB12, key controllers of cilium formation and ciliary vesicle transport [24, 25], mediate LRRK2 impact on ciliogenesis [23, 26, 27].

In this study, we demonstrate that LRRK2 is expressed in murine and human islets of Langerhans and in clonal β-cells where it regulates insulin secretion affecting primary cilium formation and RAB8 phosphorylation. Overall, our results identify for the first time LRRK2 and RAB8 as new players in the context of insulin release and ciliogenesis in pancreatic β-cells.

## Materials and methods

### Cell culture and transfection

Mouse βtc3 cell line was kindly provided by Prof. Douglas Hanahan (Department of Biochemistry and Biophysics, University of California, San Francisco, CA). βtc3 cells were grown in RPMI 1640 medium (Euroclone, Cat. ECB9006L) supplemented with 10% heat-inactivated fetal bovine serum (FBS – Euroclone, Cat. ECS5000L), 1% Glutamine (Euroclone, Cat. ECB3000D), 1% penicillin-streptomycin (Euroclone, Cat. CB3001D). βtc3 cells were transiently transfected with LRRK2 full-length, LRRK2 G2019S, WT RAB8 and T72A mutants (25–30 µg), Silencer™ Negative Control (Thermo Fisher Scientific; Cat. AM4611) and siRNA RAB8A 0.6 µM (Thermo Fisher Scientific, Cat. 4390TT1) by electroporation using the Bio-Rad Gene Pulser II. Transfected cells were processed 48 h post-transfection. The full-length RFP tagged wild-type and G2019S hLRRK2 variants [28] were a kind gift from Dr. Johannes Gloeckner (DZNe, Tubingen, Germany) and were created via site-directed mutagenesis using the QuikChange mutagenesis kit (Stratagene, Cat. 200518). The RAB8a-WT and RAB8a-T72A constructs were a kind gift from Prof. Sabine Hilfiker (Rutgers New Jersey Medical School, Newark, NJ 07103, USA) [29]. EGFP-Insulin was a kind gift from Dr. Rustenbeck (Technische Universität Braunschweig, Germany) [30]. The two LRRK2 inhibitors were used as follow: *rel*−3-[6-[(2*R*,6*S*)−2,6-Dimethyl-4-morpholinyl]−4-pyrimidyl]−5-[(1-methylcyclopropyl) oxy]−1*H*-indazole (MLi-2; 10 nM, 45–60 min; Tocris, Cat.5756) and 5-(2-Fluoro-4-pyridinyl)−2-(phenylmethoxy)-*N*−3-pyridinylbenzamide (GSK2578215A; 200 nM, 45–60 min; Tocris, Cat. 4629). DMSO (Merck, Cat. 276855) treated βtc3 cells or human islets were used as controls.

## Animals

Animal protocols were approved by the University of Trento and National Ministry of Health (IACUC 793/2016-PR). The G2019S-LRRK2 BAC mice [31] were backcrossed onto the C57BL/6J mice for 10 generations. Animals had free access to food and were kept in a normal light/dark cycle (12 h light:12 h dark). Fasting conditions lasted 6 h during the light cycle. Only male mice (12 and 18 months of age) were considered for the study due to a gender effect.

## Mouse islets isolation

Pancreata of C57BL/6J wild-type mice or G2019S-LRRK2 BAC mice were perfused with Hanks’ Balanced Salt 1x solution (HBSS) containing 2 mg/mL of collagenase from Clostridium histolyticum (Merck, Cat. C9263) and digested at 37 °C for 17.5 min. After centrifugation, the islets were isolated on a gradient of Histopaque 1119 (Merck, Cat. 11191), Histopaque 1077 (Merck, Cat. 10771), and HBSS 1x containing 0.4% BSA (Merck, Cat. A8022) [32]. Handpicked islets from three distinct animals were pooled and plated in 96-well plates (30 islets/well) for insulin secretion measurement and cultured in RPMI 1640 supplemented with 10% heat-inactivated FBS, 1% Glutamine and 1% penicillin-streptomycin in humidified atmosphere containing 5% of CO_2_ at 37 °C.

## Human islets preparations

De-identified human primary islets isolated from deceased donors were obtained from Niguarda Ca’ Granda hospital (Milan, Italy) according to the procedure described [33] in conformity to the ethical requirements approved by the Niguarda Cà Granda Ethics Board. The human islets (80 ± 10% purity) were cultured in RPMI culture medium containing 5.5 mmol/L glucose (Merck, Cat. R1383), 10% heat-inactivated FBS, 0.7 mM glutamine and 50 units/mL penicillin-streptomycin for 3 days [34].

## Reverse Transcriptase-PCR

Expression of LRRK2 protein in the endocrine pancreas was confirmed by reverse transcriptase-PCR using the MultiScribe™ Reverse Transcriptase (Invitrogen, Cat. 4311235). Total RNA from 9 × 10^6^ βtc3 or 1500 isolated human islets of Langerhans was extracted with Trizol (Invitrogen, Cat. 15596-026). For cDNA synthesis, 2 µg of digested RNA was reverse transcribed using random oligonucleotides (final concentration, 12.5 ng/µL) as primers and 200 Moloney murine leukaemia units. Pfu Polymerase (Promega, Cat. M7741) was used as DNA polymerase. To confirm the absence of genomic contamination in the RNA samples, reverse transcriptase negative controls were introduced in each experiment (without Moloney murine leukaemia virus reverse transcriptase). Primer sequences for human: forward 5’-TGGGAAATACTGGGAGTGGT-3’ and reverse 5’-ACCTGCAAAATCCCACACAT-3’. Primer sequences for mouse: forward 5’-CGTCCTCGGATGTTGGTAAT-3’ and reverse 5’-TAGTCCGCAATCTTCGCAAT-3’ [32].

### Vesicle trafficking by acridine orange assay

βtc3 cells were loaded with 100 nM Acridine Orange (Merck, Cat. A6014) in Krebs-Ringer-HEPES (KRH) buffer supplemented with 1 mM glucose at 37 °C for 15 min. Then cells were rinsed and incubated for 45 min in KRH buffer with or without the specific LRRK2 inhibitors (10 nM MLi-2 or 200 nM GSK), both under basal (1 mM glucose) and stimulated conditions (20 mM glucose or 40 mM KCl). At the end of the incubation, the fluorescence intensity of the medium was measured with the microplate TECAN infinite F500 reader (535/590 nm Ex/Em).

## Insulin secretion

βtc3 cells, isolated mouse- or human-islets were seeded in 96-well plate. The cells or islets (40 islets/well) were maintained in KRH 1 mM glucose for 1 h and then they were transferred to low-glucose (1 mM for βtc3 cells; 3 mM for mouse and human islets) or high-glucose (20 mM for βtc3 cells; 28 mM for mouse islets; 16.7 mM for human islets) KRH buffer plus 0.2% BSA in the presence or absence of LRRK2 inhibitors (10 nM MLi-2 or 200 nM GSK). After 45 min of static incubation, the culture supernatants were collected. Insulin content and secretion were measured by ELISA following the manual of the manufacturer (Mercodia, Cat. 10-1113-01 and Cat. 10-1247-01). For the detection of insulin in serum, blood samples were collected and centrifuged for 14 min at 4000 g at 4 °C. The serum insulin concentration was detected by ELISA assay (Mercodia, Cat. 10-1247-01) [32].

## Pull-down assay

6 × 10^6^ βtc3 cells were transfected with full length RFP-LRRK2 (30 µg) and pull-down assay was performed 48 h after transfection. Cells were lysed in 250 µL of lysis buffer (150 mM NaCl, 20 mM Tris-HCl pH 7.4, 1 mM EDTA 1 mM, 1% Tween 20) supplemented with aprotinin (Merck, Cat. A4529), PMSF (Merck, Cat. P7626), Roche inhibitors (Merck, Cat. 11873580001), sodium pyrophosphate (2.5 mM) and ortavanadate (1 mM) for 15 min at 4 °C. Control-selector (IBA Lifesciences, Cat. 2-9101-004) and RFP-selector (IBA Lifesciences, Cat. 2-9141-020) resins were pre-equilibrated with lysis buffer without inhibitors and then incubated with lysates for 2 h at 4 °C. After incubation, all samples were centrifuged for 1 min at 1000 g at 4 °C and the non-bound fractions were collected. Control-selector and RFP-selector resins were then repeatedly washed with different wash buffers (500 mM, 300 mM or 150 mM NaCl, 20 mM Tris-HCl pH 7.4, 1%, 0.5%, 0.1% or 0.02% Tween 20). Each selector resin was resuspended in β-mix, boiled at 95 °C for 5 min and centrifuged for 1 min at 3000 g at 4 °C. Bound fractions were then collected.

### Immunoblot assay

Total cytosolic protein extracts were obtained by lysing cells in 100 µL of RIPA buffer supplemented with aprotinin (Merck, Cat. A4529), PMSF (Merck, Cat. P7626) and Roche inhibitors (Merck, Cat. 11873580001) for 15 min at 4 °C. Protein concentration was evaluated by Bradford assay (Merck, Cat. B6916) and 30 µg of proteins were resolved by 9% SDS-PAGE under reducing conditions and transferred onto nitrocellulose membranes (Merck, Cat. IPVH00010). Subsequently, membranes were blocked in Tris-Buffered Saline (150 mM NaCl, 20 mM Tris-HCl pH 7.4) plus Tween 0.1% (TBS-T) containing 5% or 3% non-fat dried milk and probed with primary antibodies: mouse anti-β actin (1:10000; Novus, Cat. NB600-501), rabbit anti-LRRK2 (1:500; Abcam, Cat. ab133474), rabbit anti-RAB8 (1:1000; Abcam, Cat. ab133474), rabbit anti-RAB8 (phospho T72) (1:1000; Abcam, Cat. ab230260). Protein detection was performed using the HRP-conjugated secondary antibodies: anti-mouse (Cell Signaling, Cat. 7076 S) or anti-rabbit (Cell Signaling, Cat. 7074 S) IgGs HRP-linked and LiteUP WB Chemiluminescent Substrate (Euroclone, Cat. EMP002005). Protein band density was quantified by Image Studio™ Lite software (Li-Cor, Biosciences) [35].

### Immunofluorescence staining

Glass coverslip grown βtc3 cells were fixed in 4% paraformaldehyde (PFA - Merck, Cat. 818715) for 20 min and labelled with guinea pig anti-insulin polyclonal (1:250; Dako, Cat. A0564), rabbit anti-LRRK2 (1:50; Abcam, Cat. ab133474), rabbit anti-RAB8 (1:200, Abcam, Cat. ab133474), rabbit anti-RAB8 (phospho T72) (1:200, Abcam, Cat. ab230260), mouse anti-acetylated tubulin (1:300, Merck, Cat. T7451) as previously described [36]. For morphological studies, pancreases from wild type and transgenic mice were fixed in 4% neutral-buffered formalin, processed, and embedded in paraffin blocks. After microwave antigen retrieval with 10 mM citrate buffer (pH 6.0), 5-µm-thick sections were incubated with primary antibodies against hormones: guinea pig anti-insulin polyclonal (1:350; Dako, Cat. A0564), mouse anti-glucagon polyclonal (1:50; R&D Systems, Cat. MAB1249), rat anti-somatostatin monoclonal (1:75; Merck, Cat. MAB354), mouse anti-acetylated tubulin (1:300; Sigma, Cat. T7451). The following secondary antibodies were employed: rhodamine-conjugated anti-guinea pig IgG (1:150; Cat. 706-295-148, Jackson ImmunoResearch Laboratories), FITC-conjugated anti-rat IgG (1:150; Cat. 112-095-068, Jackson ImmunoResearch Laboratories), FITC-conjugated anti-rabbit IgG (1:150; Cat. 111-095-006, Jackson ImmunoResearch Laboratories), Cy5-conjugated anti-mouse IgG (1:150; Cat. 715-175-151, Jackson ImmunoResearch Laboratories), goat anti-mouse IgG (1:150; Invitrogen, Cat. A11029). Immunofluorescence microscopic analysis was performed using the Axio Observer Z1 microscope (Zeiss) equipped with a 40x or 100x (mouse sections) or 100x (βtc3 cells) objective. Identical parameters (acquisition time and gain) were maintained to acquire images in the distinct samples. Image-Pro Plus Analyzer Image Software (Media Cybernetics, Bethesda, MD, USA) was used to analyze all images acquired.

*Quantification of ciliated cells.* The percentage of ciliated cells was evaluated in serum-fed (cells maintained in complete medium and then fixed) and serum-starved (cells maintained in medium w/o serum for 60 min and then fixed) conditions. Mean cilia prevalence was assessed by counting the number of primary cilia (positive for acetylated α-tubulin) over the total number of cells (positive for DAPI) in randomly selected fields, blinded by two independent observers (20 to 50 fields analysed per condition).

*Co-localization analysis.* The Pearson’s coefficient between RAB8 and acetylated α-tubulin or LRRK2 and insulin stainings was assessed using the Image-Pro Plus Co-localisation plug-in within the region of interest (ROI) outlined on the ciliary structure or on the cell area (insulin/LRRK2).

### Total internal reflection fluorescence microscopy (TIRFM)

Samples were imaged by a TIRF microscope (Axio Observer Z1, Zeiss), equipped with a 100 × 1.45 NA, oil immersion, objective and an Argon laser.

For insulin granules count, cells were fixed in 4% PFA, labelled with the guinea pig anti-insulin antibody (1:350; Dako, Cat. A0564) and imaged by TIRFM. Green fluorescence was excited using the 488-nm laser line and acquired with a band-pass filter (Zeiss) mounted on a Retiga SRV CCD camera [32]. The ImageJ particle analysis plug-in was employed to quantify the number of fluorescent objects. The area (µm^2^) of the cells and the number of insulin-positive granules per cell were measured in a software-assisted manner. Collected data were normalized to cell area.

For insulin and LRRK2 colocalization, cells were stained with guinea pig anti-insulin polyclonal (1:350; Dako, Cat. A0564) and rabbit anti-LRRK2 (1:50; Abcam, Cat. ab133474) antibodies. Green and red fluorescence were excited using the 488-nm and 535-nm laser line, respectively and acquired with a band-pass filter (Zeiss) mounted on a Retiga SRV CCD camera. The Pearson’s coefficient between LRRK2 and insulin was assessed using the Image-Pro Plus Co-localisation plug-in within the region of interest (ROI) outlined on the cell area (insulin/LRRK2).

For time lapse experiments, cells were transfected with the GFP-insulin construct and RAB8 WT or mutants and 48 h after transfection, single-cell imaging under TIRF illumination using the 488 nm laser line was performed at 2 Hz, for a total of thirty seconds, at different times after perfusion with 20 mM glucose or 40 mM KCl KRH at 25 °C. After deconvolution and photobleaching correction, images were analyzed using an existing Image-Pro Plus plug-in (tracking object) for selection and quantification of fluorescent spots (Media Cybernetics, Bethesda, MD, USA). The following criteria were used to select the individual spots: mean area (0.039-1 µm^2^), minimal pixel intensity (3 x average background), aspect score (1–3). On each coverslip, up to five cells were imaged in at least three independent experiments for each construct and data were normalized to cell area.

### Statistical analysis

All statistical analyses were performed with GraphPad Prism 9.0 on independent biological replicates. Data are presented as means ± S.D. The number of replicates for each experiment is reported in the figure legend. Statistical comparisons were performed by unpaired Student’s t-test or analysis of variance (ANOVA) followed by Tukey’s multiple comparison test. A P value < 0.05 was considered statistically significant.

## Results

### LRRK2 G2019S mice exhibit altered metabolic profile and impaired insulin secretion

**Fig. 1:**
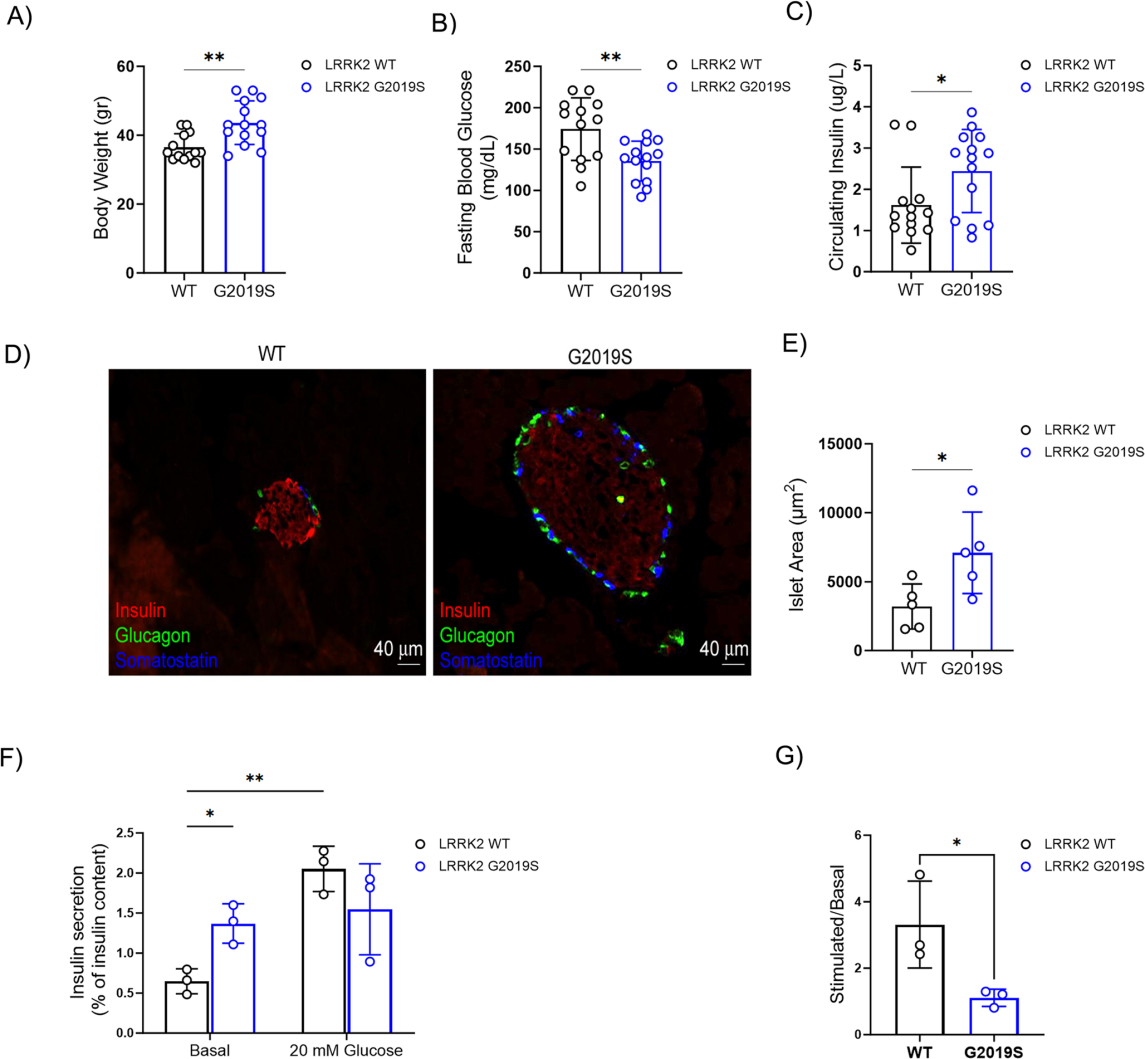
LRRK2 G2019S mice exhibit altered metabolic profile and impaired insulin secretion. **A-C** Quantitative analysis of **A** body weight (gr), **B** blood glucose (mg/dL), and **C** circulating insulin (µg/L) in LRRK2 WT and G2019S mice (*n* = 13 WT and 14 G2019S mice). Blood glucose and insulin levels were measured under fasting conditions (6 h, light cycle). Data are reported as mean ± SD. Unpaired Student’s T-test: *p < 0.05; ** *p* < 0.01. **D** Representative immunofluorescence images of islets of Langerhans from LRRK2 WT and G2019S mice stained with anti-insulin (red), anti-glucagon (green), and anti-somatostatin (blue) antibodies. Scale bar: 40 μm. **E** Size area (µm^2^) of islets of Langerhans from LRRK2 WT and G2019S mice (*n* = 5 mice/group). Data are reported as mean ± SD. Unpaired Student’s T-test: *p < 0.05. **F** Glucose-stimulated (20 mM) insulin secretion in islets of Langerhans isolated from LRRK2 WT and G2019S mice (*n* = 3 independent experiments in triplicate). Data are reported as mean ± SD. Two-way ANOVA: *p < 0.05, **p < 0.01. **G** Stimulatory index of insulin secretion. Data (mean ± SD) are expressed as stimulated over basal release (*n* = 3 independent experiments in triplicate). Unpaired Student’s T-test: * *p* < 0.05

Recent data involves LRRK2 in glucose homeostasis [10], therefore, we characterized the metabolic profile of aged hLRRK2 G2019S BAC mice (> 12 months, hereinafter G2019S mice). These mice develop an age-related decrease in striatal dopamine content, and behavioral motor deficits [31, 37]. Compared to the WT group, the G2019S mice exhibited several metabolic defects, including a significant body weight increment (Fig. 1A), lower glycaemia (Fig. 1B), and higher plasma insulin levels (Fig. 1C) under fasting conditions (6 h, light cycle). Hence, we used the homeostasis model assessment of β-cell function (HOMA-β) and insulin resistance (HOMA-IR) to evaluate if the metabolic defects were the result of an altered β-cell functionality and/or insulin action (Suppl. Fig S1A and S1B). Analyses of indexes indicate that the observed phenotype was associated to higher β-cell activity (HOMA-β) in G2019S mice compared to WT mice. These observations prompted us to investigate whether *LRRK2* G2019S mutant could affect insulin production and/or pancreatic β-cell function. We therefore looked at pancreatic islet morphology. The triple immunofluorescence staining of islets of Langerhans from WT and *LRRK2* G2019S mice (Fig. 1D) revealed consistent differences with significantly larger islets in the pancreas of G2019S mice (8088 ± 680 µm^2^) compared to WT mice (3195 ± 328 µm^2^) (Fig. 1E).

To directly assess the islet-intrinsic role of LRRK2, we isolated pancreatic islets from WT and G2019S mice and then we evaluated the basal and stimulated insulin release by ELISA assays. Compared to the control group, G2019S mice exhibited significantly higher basal insulin secretion, but no further increase was observed after glucose stimulation (Fig. 1F). Thus, the stimulated ratio (stimulated/basal insulin secretion) observed was significantly lower in G2019S mice compared to WT littermates (Fig. 1G).

Taken together, these observations suggest that alterations in LRRK2 kinase activity are extremely relevant to pancreatic β-cell function in vivo and that the hyperinsulinaemic phenotype observed in the G2019S mice might be a result of improper insulin production and/or release.

### LRRK2 is expressed in the endocrine pancreas and is involved in insulin release in βtc3 cells

**Fig. 2:**
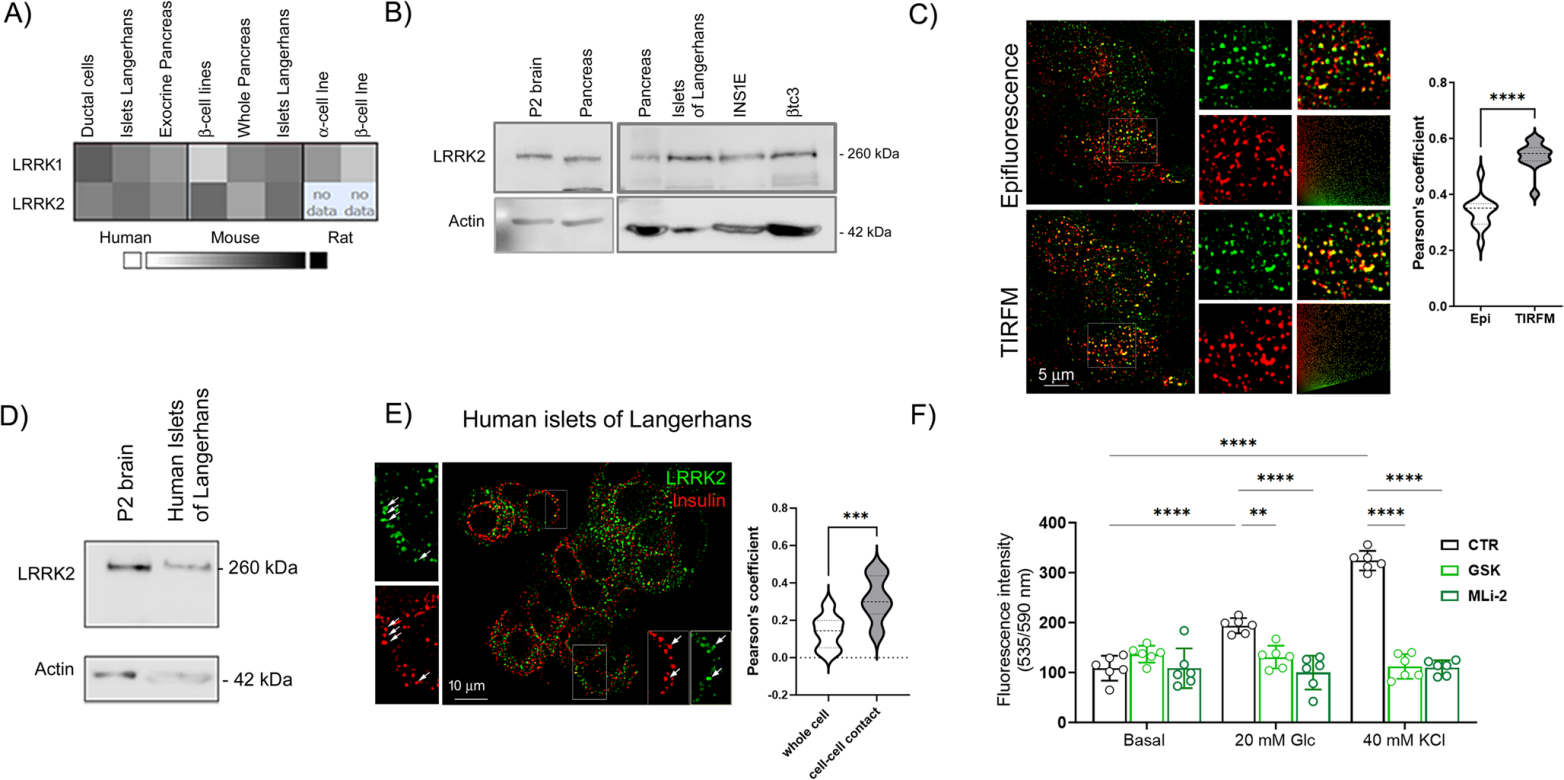
LRRK2 is expressed in the endocrine pancreas and is involved in insulin release in βtc3 cells. **A** Expression of LRRK2 gene in human, mouse, and rat pancreas according to the T1D Protein Atlas database http://T1DBase.org [38]. **B** Western blot of LRRK2 expression in murine pancreas, isolated murine islets of Langerhans, and rodent β-cell lines (βtc3 and INS1E). Mouse P2 brain fraction was used as positive control. Actin was used as loading control. **C** Representative immunofluorescence images of βtc3 cells double stained with anti-insulin (red) and anti-LRRK2 (green) antibodies visualized by epifluorescence and TIRFM. The colocalization between LRRK2 and insulin is shown in yellow. Scale bar: 5 μm. Particulars are shown at higher magnification (3X). The Pearson’s coefficient of colocalization between insulin and LRRK2 in epifluorescence and TIRFM images is reported (*n* = at least 12 images). Data are reported as mean ± SD. Unpaired Student’s T-test: ****p < 0.001. **D** Western blot of LRRK2 expression in isolated human islets. Mouse P2 brain fraction was used as positive control. Actin was used as a loading control. **E** Representative immunofluorescence image of a human islet double stained with anti-insulin (red) and anti-LRRK2 (green) antibodies. Colocalization is shown in yellow. Scale bar: 10 μm. Particulars are shown at higher magnification (2X), arrows indicate superimposable vesicles. The Pearson’s coefficient of colocalization between insulin and LRRK2 in ROIs located on the whole insulin-positive cell or at cell-cell contact sites is reported (*n* = 24 ROIs in 4 different islet images). Data are reported as mean ± SD. Unpaired Student’s T-test: ***p < 0.005. **F** Vesicle trafficking under basal (1 mM glucose) and stimulated (20 mM glucose or 40 mM KCl) conditions was evaluated in βtc3 cells in the absence (CTR - DMSO) or presence of LRRK2 kinase activity inhibitors (200 nM GSK and 10 nM MLi-2) by using an acridine orange assay (*n* = 6 independent experiments). Data are reported as mean ± SD. Two-way ANOVA: **p < 0.01; ****p < 0.001

Outside the brain, LRRK2 has been found in several tissues and there is compelling evidence that the kinase is expressed in both human and rodent pancreas, with an enrichment in the endocrine pancreas [38] (Fig. 2A). We have confirmed the expression of LRRK2 protein in the mouse pancreas and in isolated pancreatic islets as well as in the rodent β-cell lines βtc3 and INS1E (Fig. 2B). We detected LRRK2 expression at the mRNA level in βtc3 cells using RT-PCR (Suppl Fig S2A). Double-staining immunofluorescence experiments performed on βtc3 cells using anti-insulin (red) and anti-LRRK2 (green) antibodies showed that LRRK2 partially colocalized with vesicular structures positive for insulin (Pearson’s coefficient 0.34 ± 0.06) (Fig. 2C). Interestingly, the colocalization was significantly increased (Pearson’s coefficient 0.54 ± 0.05) when cells were imaged by TIRFM, which allows the selective visualization of insulin granules close to the plasma membrane (Fig. 2C). Furthermore, we identified LRRK2 expression at the mRNA and protein levels in human islets of Langerhans (Suppl Fig S2B and Fig. 2D). Double immunofluorescence staining revealed the presence of LRRK2 in close proximity to vesicular structures both in insulin-positive and -negative cells of the islet. A colocalization analysis performed on different regions of the islet revealed increased colocalization between the two stainings at cell-cell contacts (Pearson’s coefficient 0.30 ± 0.11), where insulin granules fuse with the plasma membrane, compared to the cell center (Pearson’s coefficient 0.13 ± 0.09) (Fig. 2E).

Cumulating observations indicate that LRRK2 kinase activity influences vesicle trafficking [22, 39–41]. Herein, we first explored if LRRK2 kinase activity impacts on vesicles trafficking in βtc3 cells, taking advantage of two potent and specific LRRK2 inhibitors, GSK2578215A (hereinafter GSK) [42] and MLi-2 [43]. Incubation of β-cells with both inhibitors did not change the LRRK2 protein expression nor affect cell viability (Suppl Fig S2C and S2D). We tracked vesicles by loading the cells with acridine orange, a pH-sensitive dye that accumulates in acidic vesicular structures and is released upon vesicles fusion, and we monitored exocytosis under basal and stimulated conditions. As expected, incubations with both 20 mM glucose and 40 mM KCl (a depolarizing stimulus) resulted in an increase of the fluorescent signal in the medium (Fig. 2F). LRRK2 inhibitors did not alter basal release but prevented the fluorescence increase observed upon both stimulations (Fig. 2F). Overall, these data suggest that LRRK2 kinase activity may control stimulated vesicular trafficking, which is mainly implicated in insulin secretion in β-cells.

### LRRK2 controls glucose-stimulated insulin secretion through its kinase activity

**Fig. 3:**
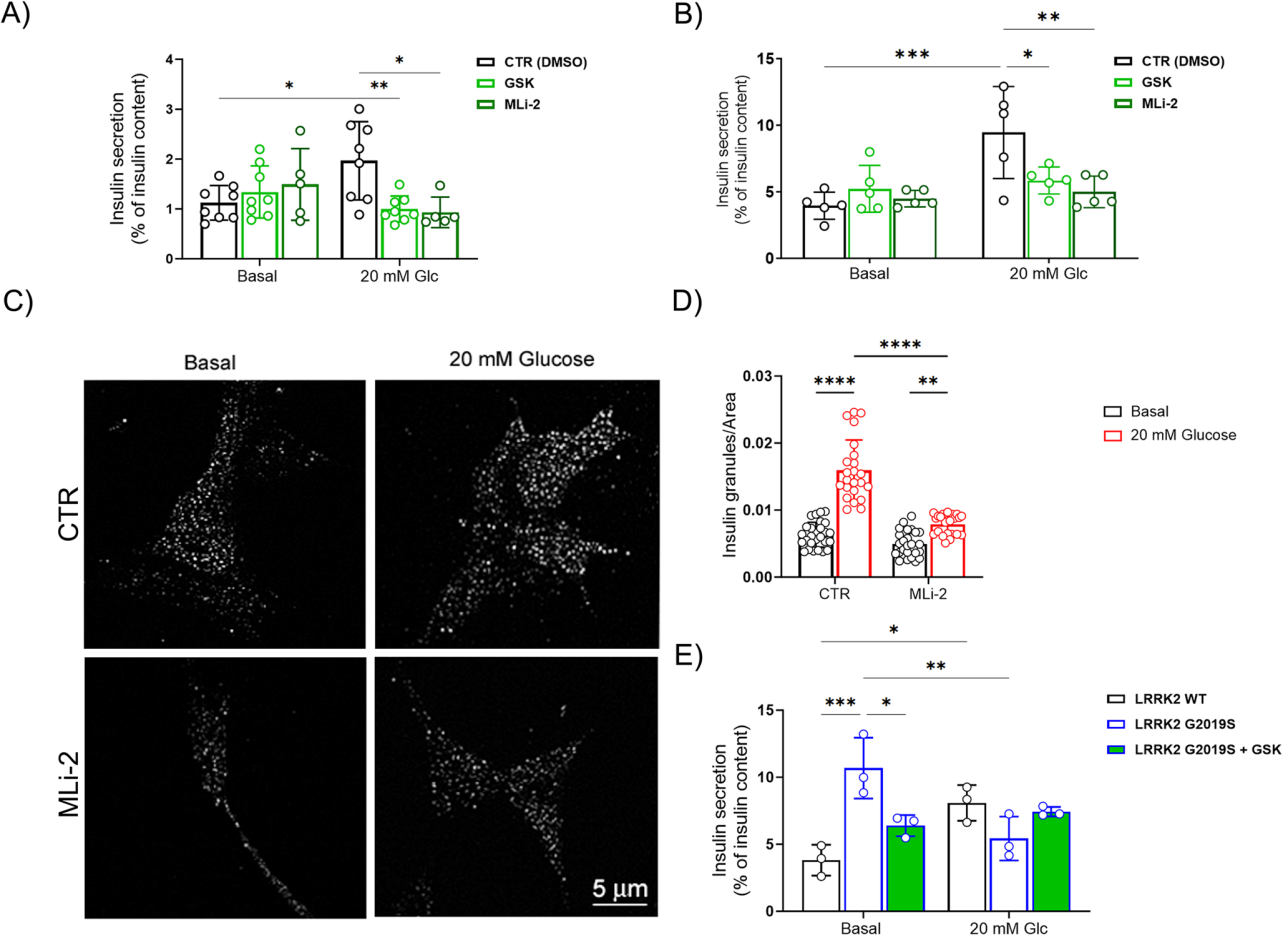
LRRK2 controls the glucose-stimulated insulin secretion through its kinase activity. **A** Glucose-stimulated (20 mM) insulin secretion in βtc3 cells incubated in the absence (CTR - DMSO) or presence of the LRRK2 kinase inhibitors GSK (200 nM) or MLi-2 (10 nM) (n = at least 5 independent experiments). Data are expressed as percentage of insulin content and are reported as mean ± SD. Two-way ANOVA: * *p* < 0.05, **p < 0.01. **B** Glucose-stimulated (16.7 mM) insulin secretion in isolated human islets incubated in the absence (CTR - DMSO) or presence of the LRRK2 kinase inhibitors GSK (200 nM) and MLi-2 (10 nM) (*n* = 5 independent experiments). Data are expressed as percentage of insulin content and are reported as mean ± SD. Two-way ANOVA: *p<0.05, **p < 0.01, ***p < 0.005. **C** Representative images of insulin granules density in the TIRF zone (100 nm) under basal (1 mM glucose) and stimulated (20 mM glucose) conditions in βtc3 cells incubated with 10 nM MLi-2 or DMSO (CTR) for 45 min. After treatments, cells were fixed and stained with anti-insulin antibody. Scale bar: 5 μm. **D** Quantitative analysis of insulin granules in the TIRF zone in control and MLi-2 treated βtc3 cells. Data are normalized for the cell area and are reported as mean ± SD. Each dot represents the average granule density in one cell (*n* = 22 cells). Two-way ANOVA: **p < 0.01, ****p < 0.001. **E** Glucose-stimulated (20 mM) insulin secretion in βtc3 cells expressing LRRK2 WT or G2019S constructs. Insulin secretion was evaluated in the presence or absence of the LRRK2 kinase inhibitor GSK (200 nM) in G2019S-transfected cells (*n* = 3 independent experiments). Data are expressed as percentage of insulin content and are reported as mean ± SD. Two-way ANOVA: *p < 0.05, **p < 0.01, ***p < 0.005

These findings prompted us to investigate the effects of LRRK2 kinase inhibitors on the hormone secretion. We monitored insulin in the media by means of ELISA assays. The 200 nM GSK or 10 nM MLi-2 treatments did not alter basal insulin secretion but significantly prevented the glucose-stimulated release in both βtc3 cells (Fig. 3A) and human isolated islets (Fig. 3B). These observations reinforce the hypothesis that LRRK2 kinase activity is critical for the control of the glucose-stimulated insulin secretion (GSIS).

To confirm the possible impact of LRRK2 kinase activity on insulin granules trafficking/fusion, we exploited TIRF microscopy and monitored the localization of insulin granules at the plasma membrane in βtc3 cells upon treatment with 10 nM MLi-2. In absence of any stimulation, the distribution of insulin-positive granules in the TIRF zone was similar between control and MLi-2-treated cells (Fig. 3C and D). After glucose stimulation, the density of insulin granules at the plasma membrane was significantly increased in control cells, whereas this redistribution was almost prevented in MLi-2 treated cells (Fig. 3C and D).

To complement this data, we investigated the impact of increased LRRK2 kinase activity. Since *LRRK2* G2019S pathogenic mutation is associated with enhanced kinase activity, we evaluated insulin secretion in βtc3 cells transfected with LRRK2 WT or G2019S mutant. The overexpression of WT LRRK2 did not change the basal and stimulated insulin release compared to cells transfected with an empty vector (Suppl Fig S3). LRRK2 G2019S transfected cells exhibited increased insulin release already at baseline compared to the WT group, whereas no further increase in insulin secretion was observed after high glucose (20 mM) stimulation (Fig. 3E), corroborating the results obtained in islets isolated from G2019S BAC mice. We also found that 200 nM GSK treatment reversed the phenotype in LRRK2 G2019S transfected cells (Fig. 3E).

All together, these data indicate that LRRK2 kinase activity may promote the stimulated insulin release by controlling the mobilization of insulin granules.

### RAB8 is phosphorylated by LRRK2, and its phosphorylation promotes insulin release

**Fig. 4:**
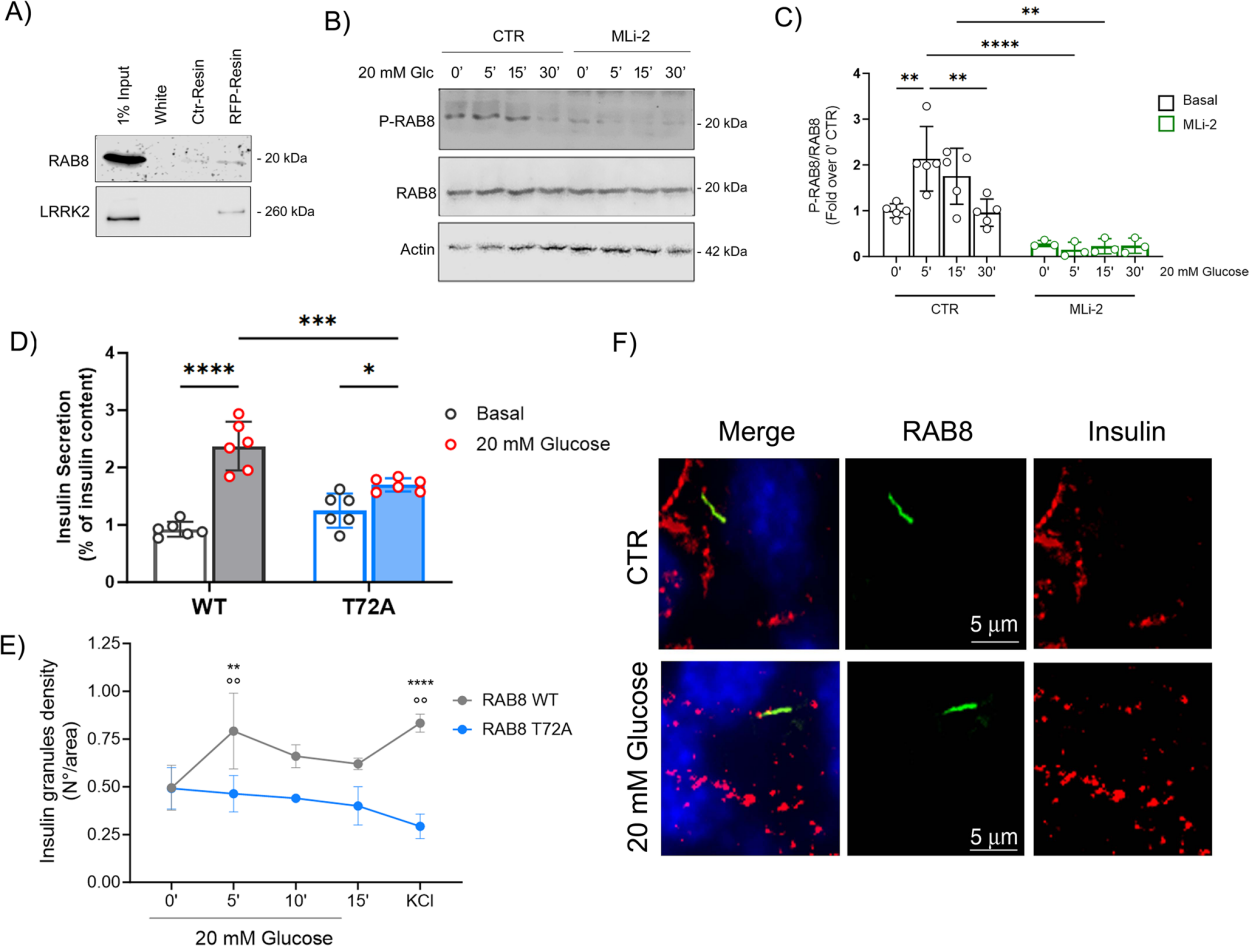
RAB8 is phosphorylated by LRRK2, and its phosphorylation promotes insulin release. **A** Full-length RFP-LRRK2 was expressed in βtc3 cells, and the recombinant protein was isolated on the RFP-selector resin (RFP-resin). A control-selector resin (Ctr-Resin) was used to detect unspecific binding. Interacting proteins were resolved by western blotting analysis with the anti-RAB8 antibody. **B** Western blot analysis of RAB8 phosphorylation on the threonine 72 residue at different time points after glucose stimulation (20 mM), in the presence or absence of LRRK2 kinase inhibitor MLi-2 (10 nM, 45 min pre-treatment) and **C** relative quantification (*n* = at least 3 independent experiments). Actin was used as loading control. Data are expressed as Phospho-RAB8 over RAB8 and are reported as mean ± SD. Two-way ANOVA: **p < 0.01; ****p < 0.001. **D** Glucose-stimulated (20 mM) insulin secretion in βtc3 cells transfected with WT RAB8 and T72A mutant. Data are expressed as percentage of insulin content and values are reported as mean ± SD (*n* = 6 experiments). Two-way ANOVA: *p < 0.05; ***p < 0.005; ****p < 0.001. **E** Quantitative analysis of insulin granule trafficking in the TIRF zone in βtc3 cells cotransfected with GFP-insulin and WT RAB8 and T72A mutant at different time points after glucose (20 mM) and KCl (40 mM) stimulations. Data are normalized on cell area and are reported as mean ± SD. Two-way ANOVA. **p < 0.01; ****p < 0.001 vs WT RAB8, same time point; °°*p* < 0.01, vs 0’ WT RAB8 (*n* = at least 3 independent experiments). **F** Representative immunofluorescence images of βtc3 cells triple stained with DAPI (blue), anti-RAB8 (green) and anti-insulin (red) antibodies under basal (1 mM glucose) and stimulated (20 mM glucose) conditions. Scale bar: 5 μm

To gain a better understanding of the molecular mechanisms underlying LRRK2 action in β-cells, we investigated the LRRK2 interactome. In particular, we performed pull down experiments on βtc3 cells transfected with the full length LRRK2 construct, revealing that RAB8, a small GTP-binding protein involved in the regulation of vesicular trafficking and a well-known physiological substrate of LRRK2 [44], is an effective binding partner of LRRK2 in β-cells (Fig. 4A).

Phosphoproteomic studies have shown that LRRK2 phosphorylates RAB8 on threonine 72, a residue located in a region involved in interaction with the regulatory proteins [45, 46]. We monitored the RAB8 T72 phosphorylation profile over time after the exposure to high glucose (20 mM) and observed a transient increase in RAB8 phosphorylation, which peaked at 5 min and declined after 30 min of treatment (Fig. 4B and C). This transient glucose-stimulated RAB8 phosphorylation was LRRK2-dependent, as it was completely prevented by treatment with 10 nM MLi-2 (Fig. 4B and C).

To elucidate the potential involvement of the LRRK2-RAB8 axis in the GSIS, we took advantage of phospho-null (T72A RAB8) mutant. Upon its transfection in βtc3 cells, we evaluated insulin release by means of ELISA assays. Basal and stimulated (20 mM glucose) insulin release was not altered by the overexpression of WT RAB8 (Suppl Fig S4A), while overexpression of the T72A RAB8 phospho-null mutant impaired stimulated secretion without affecting the basal one (Fig. 4D).

To complement these observations, we monitored insulin granules dynamics in GFP-insulin expressing cells by TIRFM studies. During glucose (20 mM) or KCl (40 mM) stimulation we counted the number of insulin-positive spots approaching the TIRFM zone. WT RAB8 transfected cells exhibited a first-peak increase in insulin-positive spots after 5 min of glucose-stimulation, and a further increase after KCl stimulation (Fig. 4E). T72A RAB8 transfected cells showed similar insulin granules density at the plasma membrane under basal conditions, but no further changes in the number of insulin-spots were observed upon glucose stimulation or KCl depolarization (Fig. 4E).

Taken together, these data suggest that RAB8 phosphorylation may control insulin granules trafficking toward the plasma membrane.

RABs associate with and regulate endomembrane trafficking [47]. Thus, we investigated whether RAB8 or phosphorylated RAB8 localized on insulin granules. However, double-staining immunofluorescence experiments performed on βtc3 cells using anti-RAB8/phospho-RAB8 (green) and anti-insulin (red) antibodies, did not indicate a major overlap between RAB8/P-RAB8 and insulin positive vesicles (Fig. 4F and Suppl Fig S4B). This data may indicate that, rather than influencing insulin vesicles trafficking by a direct binding, RAB8 may control GSIS via an alternative mechanism.

### LRRK2-phosphorylated RAB8 impacts primary cilia formation

**Fig. 5:**
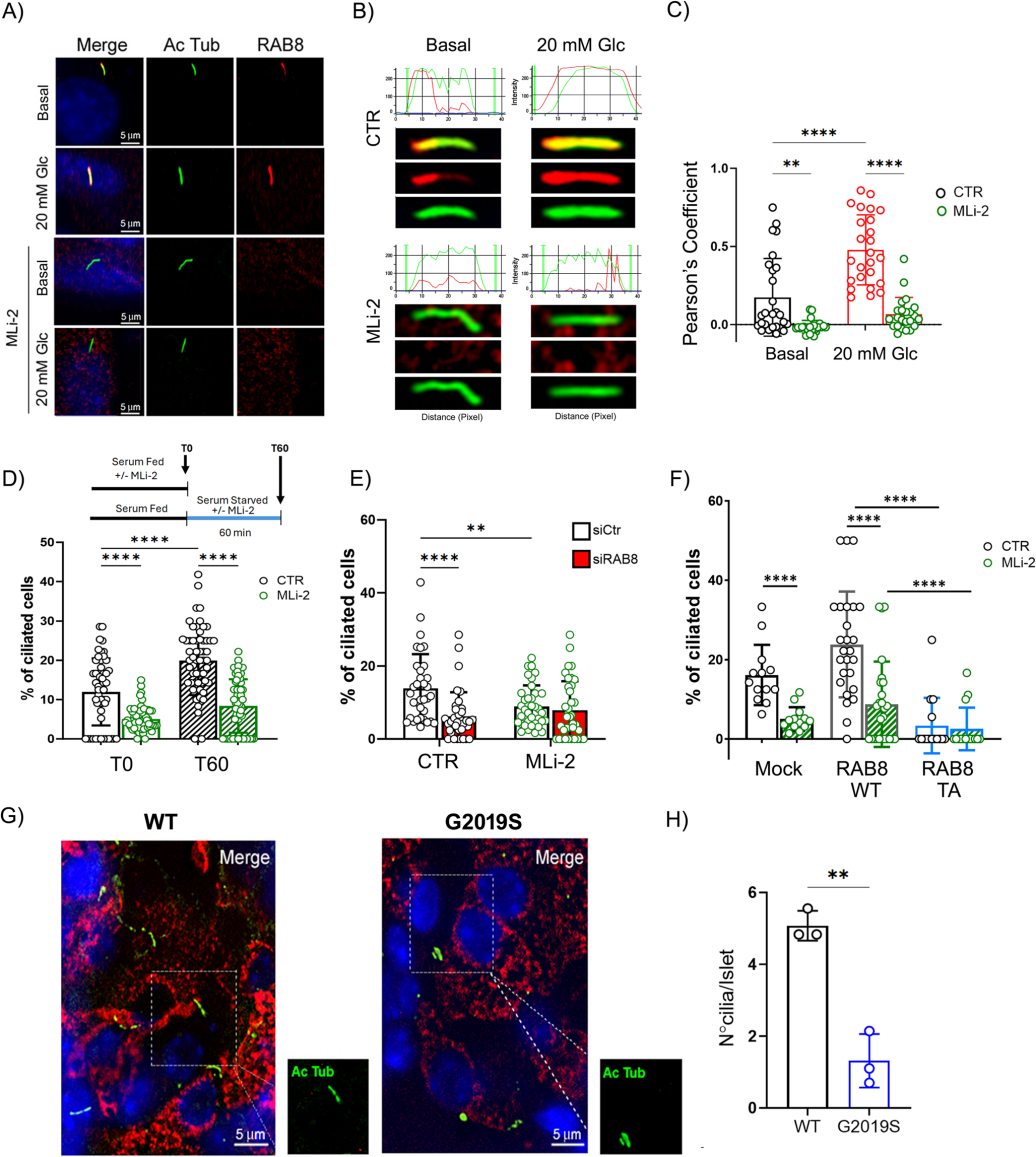
RAB8 phosphorylation impacts primary cilia formation. **A** Representative immunofluorescence images of βtc3 cells triple stained with DAPI (blue), anti-acetylated α-tubulin (green) and anti-RAB8 (red) antibodies. βtc3 cells under basal (1 mM glucose) and stimulated (20 mM glucose) conditions were incubated with/without the LRRK2 kinase inhibitor MLi-2 (10 nM; 60 min treatment). The colocalization between acetylated tubulin and RAB8 is shown in yellow/orange. Scale bar: 5 μm. **B** Fluorescent intensity profile of acetylated tubulin (green) and RAB8 (red) in the representative cilia of panel A (4x magnification). **C** The colocalization between acetylated tubulin and RAB8 was quantified by means of Pearson’s coefficient analysis (*n* = at least 23 cells per condition). Data are reported as mean ± SD. Two-way ANOVA: **p < 0.01; ****p < 0.001. **D** Quantification of ciliated βtc3 cells at 0 and 60 min after serum withdrawal, in the presence or absence of MLi-2 (10 nM for 60 min) (1 mM glucose) (n = at least 60 image fields per condition). A timeline of the treatments is depicted. Data are reported as mean ± SD. Two-way ANOVA: ****p < 0.001. **E** Quantification of ciliated βtc3 cells after RAB8 silencing using an siRNA strategy, following a 60-minute incubation in serum-starved conditions plus 1 mM glucose, in the presence or absence of MLi-2 (10 nM; 60 min treatment) (*n* = 37 image fields per condition). Data are reported as mean ± SD. Two-way ANOVA: **p < 0.01; ****p < 0.001. **F** Quantification of ciliated βtc3 cells overexpressing WT RAB8 or the T72A mutant in serum-fed and 1 mM glucose conditions in the presence or absence of MLi-2 (10 nM; 60-minute treatment) (*n* = at least 10 image fields per condition). Data are reported as mean ± SD. Two-way ANOVA: ****p < 0.001. **G** Representative immunofluorescence images of mouse islets from LRRK2 WT and G2019S mice triple stained with anti-acetylated tubulin (green), anti-insulin (red) antibodies and DAPI (blue). Scale bar: 5 μm. **H** Quantification of cilia number in islets of Langerhans from LRRK2 WT and G2019S mice (*n* = 3 mice/group; at least 12 islets per mouse). Data are reported as mean values ± SD. Unpaired Student’s T-test: ** *p* < 0.01

Several studies have demonstrated the recruitment of RAB8 to ciliary structures and documented its involvement in cilia formation and elongation [48, 49]. In β-cells the primary cilium acts as an important regulator of glucose sensing and insulin secretion [19], so we first verified the ciliary recruitment of RAB8. We identified primary cilia by localizing the acetylated α-tubulin signal and found partial colocalization with endogenous RAB8 at cilia. Interestingly, upon both glucose (Fig. 5A-C and Suppl Fig S5A) and KCl (Suppl Fig S5B and S5C) stimulation, RAB8 redistributed along the entire length of the cilium, causing a significant increase in the overlap between the two staining patterns. An anti-pThr72-RAB8 antibody revealed increased ciliary P-RAB8 staining under glucose stimulated conditions (Suppl Fig S5D). In cells treated with 10 nM MLi-2, RAB8 colocalization with acetylated tubulin dramatically decreased, regardless of glucose or KCl stimulation (Fig. 5A-C and Suppl Fig S5A, S5B and S5C). This suggests that LRRK2-mediated RAB8 phosphorylation is important for the ciliary localization of RAB8 and for its redistribution within the cilium upon glucose treatment.

Since RAB8 is a critical regulator of ciliogenesis [48, 49], we assessed whether the LRRK2 kinase activity affects cilia formation in β-cells. To induce ciliogenesis, we incubated cells in serum-free medium [50] and counted the percentage of ciliated cells before and after starvation (Fig. 5D). Under complete medium (serum-fed condition or T0), less than 12% of the cells formed cilia, whereas serum starvation for 60-min (T60 serum-starved condition) was sufficient to increase the percentage of ciliated cells to ∼20%. This increase was prevented by treatment with 10 nM MLi-2, supporting the involvement of the LRRK2 kinase activity in ciliogenesis in β-cells.

To confirm the involvement of RAB8 in this process, we silenced RAB8 using a siRNA strategy. Downregulation of RAB8 (Suppl Fig S6A) reduced the percentage of ciliated cells under both serum-starved (Fig. 5E) and serum-fed (Suppl Fig S6B) conditions. Importantly, treatment with MLi-2 did not further alter this percentage in RAB8-silenced cells, indicating that LRRK2 kinase activity affects the process by acting on RAB8 or other components of the RAB8 cascade. We confirm that RAB8 phosphorylation is important in this mechanism by analyzing the impact of the phospho-null mutant RAB8-T72A. Interestingly, the phospho-null mutant significantly reduced the percentage of ciliated cells (Fig. 5F). This reduction was not due to RAB8 overexpression, as the percentage of ciliated cells was comparable between mock cells and RAB8 WT expressing ones. Conclusively, our data suggests that cilium formation in βtc3 cells is influenced by RAB8 and, at least in part, by LRRK2-driven RAB8 phosphorylation.

Given the involvement of LRRK2 kinase in ciliogenesis in β-cells, we finally verified whether the metabolic phenotype observed in G2019S mice was associated with a cilia defect. Double immunofluorescence staining of islets of Langerhans from WT and LRRK2 G2019S mice revealed short and stubby acetylated tubulin-positive structures and a decreased number of ciliated cells in islets of Langerhans gathered from G2019S mutants compared to WT (Fig. 5G and H).

Taken together, our results indicate that the LRRK2-kinase activity modulates cilia formation in β-cells in in vitro and in vivo models.

## Discussion

This study provides evidence that LRRK2, among the major causes of familiar PD and a PD risk factor, is expressed in pancreatic β-cells and regulates the glucose stimulated insulin secretion. Mechanistically, we found that LRRK2 kinase activity controls the density of insulin granules at the plasma membrane in response to elevated glucose levels and is necessary to sustain stimulated insulin secretion. This indicates that LRRK2 kinase is temporarily activated by glucose stimulation and tunes the number of granules available for regulated secretion. We suggest that the role of LRRK2 in β-cells involves the phosphorylation of proteins controlling granules trafficking and exocytosis, as described in neurons [6, 45, 51]. This hypothesis is supported by our data showing that the ectopic expression of the hyperactive kinase G2019S LRRK2 variant in vitro promotes insulin secretion, already under basal glucose conditions. The constitutively high basal release caused by this mutant could lead to a progressive depletion of the reserve pool of granules, thereby affecting also the glucose stimulated release. Pre-treatment with the LRRK2 inhibitor may help to preserve part of this pool, thus partially rescuing the glucose stimulated release.

Interrogating the LRRK2 interactome, we found that the action of LRRK2 on insulin secretion is at least in part mediated by RAB8. RAB8, a recognized target and effector of LRRK2 in neurons [6], is a modulator of the tubulovesicular trafficking from the trans-Golgi network and recycling endosomes toward the plasma membrane [52–54]. In particular, we observed that LRRK2 binds and transiently phosphorylates RAB8 upon glucose stimulation in pancreatic β-cells. This phosphorylation is important to regulate insulin granules trafficking to the plasma membrane and insulin secretion. In fact, we detected a decreased number of insulin granules in the TIRF zone and impaired GSIS in cells expressing the T72A phospho null RAB8 mutant. Threonine 72 lies in the RAB8 switch II domain, a structural motive important to control GTP/GDP exchange and interaction with regulatory proteins [55]. In neurons, LRRK2-mediated RAB8 phosphorylation promotes the detachment from GDP dissociation inhibitors (GDIs), thus modifying the fraction of membrane-bound active RAB8 [6, 45]. RAB3 and RAB27 small GTPase proteins are known to associate with insulin granule membranes and to control late stages of exocytosis such as granules tethering at the target membrane and fusion [16, 55, 56]. Whether RAB8 may play a similar role at early steps in granule exocytosis is unknown. Of note, in neurons, LRRK2-mediated phosphorylation of RAB8 enhances its association with Rab Interacting Lysosomal Like Protein 1 and 2 (RIPL1, RIPL2) and JNK-Interacting Protein 3 and 4 (JIP3, JIP4), motor adaptor proteins involved in lysosome motility [57]. Accordingly, we may propose a direct effect of RAB8 on the movement of insulin granules from the trans Golgi network (TGN) or the recycling compartment toward the plasma membrane, along microtubules tracks, a mechanism known to sustain insulin secretion under stimulated conditions.

Although we cannot exclude that RAB8 is directly involved in insulin granules dynamics, we did not find extensive RAB8 or P-RAB8 colocalization with insulin positive structures. Clearly, the mechanism underlying LRRK2-RAB8 role on GSIS is not confined to the molecular complex controlling insulin granules exocytosis.

We focused therefore on the cilium because RAB8 has been involved in ciliogenesis [58] and primary cilia have recently been implicated in β-cell function and insulin secretion [19]. Interestingly, we found that glucose (or KCl stimulation) increases RAB8 recruitment to the cilium. Precisely, we provide evidence that glucose stimulation simultaneously triggers LRRK2-dependent RAB8 phosphorylation and the RAB8 redistribution into the cilium. Both effects are lost when LRRK2 kinase activity is inhibited, indicating the relevance of the kinase activity in this phenomenon. LRRK2 and RAB8 are critical for ciliogenesis in βtc3 cells; indeed, both pharmacological and genetic manipulation of the LRRK2-RAB8 pathway significantly change the percentage of ciliated β-cells, under both serum-fed and serum-starved conditions. Cilium formation is a complex, cellular specific, multi-step process driven by a cascade of protein activations. RAB8 has been involved in several steps, including membrane dynamics, which is fundamental for cilium initiation, and intra-flagellar transport of cargoes, which is important for cilia elongation and maintenance [49, 59]. Notably, in neurons LRRK2-mediated phosphorylation of RAB8 at T72 blocks its binding to Rabin8, required for cilium initiation, but favours its interaction with dynein and kinesin adaptors, important for intra-flagellar transport [45]. Although live-cell imaging will be necessary to better profile the specific impact of LRRK2 inhibition and hyperactivation on cilia dynamics, our work discloses a crucial involvement of LRRK2 and RAB8 in cilium physiology in β-cells.

How ciliogenesis may then modulate glucose-stimulated insulin secretion is still unclear, but a role for primary cilia in both insulin/glucose sensing and control of insulin granules exocytosis has been proposed [19, 60, 61]. In this context, it is interesting to note that decreased expression of cilia-related genes is a risk factor for the development of T2DM in rodents and humans [62]. Furthermore, ciliopathies, congenital diseases characterized by ciliary dysfunction, show metabolic abnormalities typical of T2DM. Of particular interest is Alström syndrome, which is caused by a mutation in Alms1, a gene involved in ciliogenesis. Mice carrying a mutation in the Alms1 gene are obese and hyperinsulinemic and show pancreatic islet hyperplasia [63, 64]. Strikingly, we reported a similar phenotype in the G2019S mice.

**Fig. 6:**
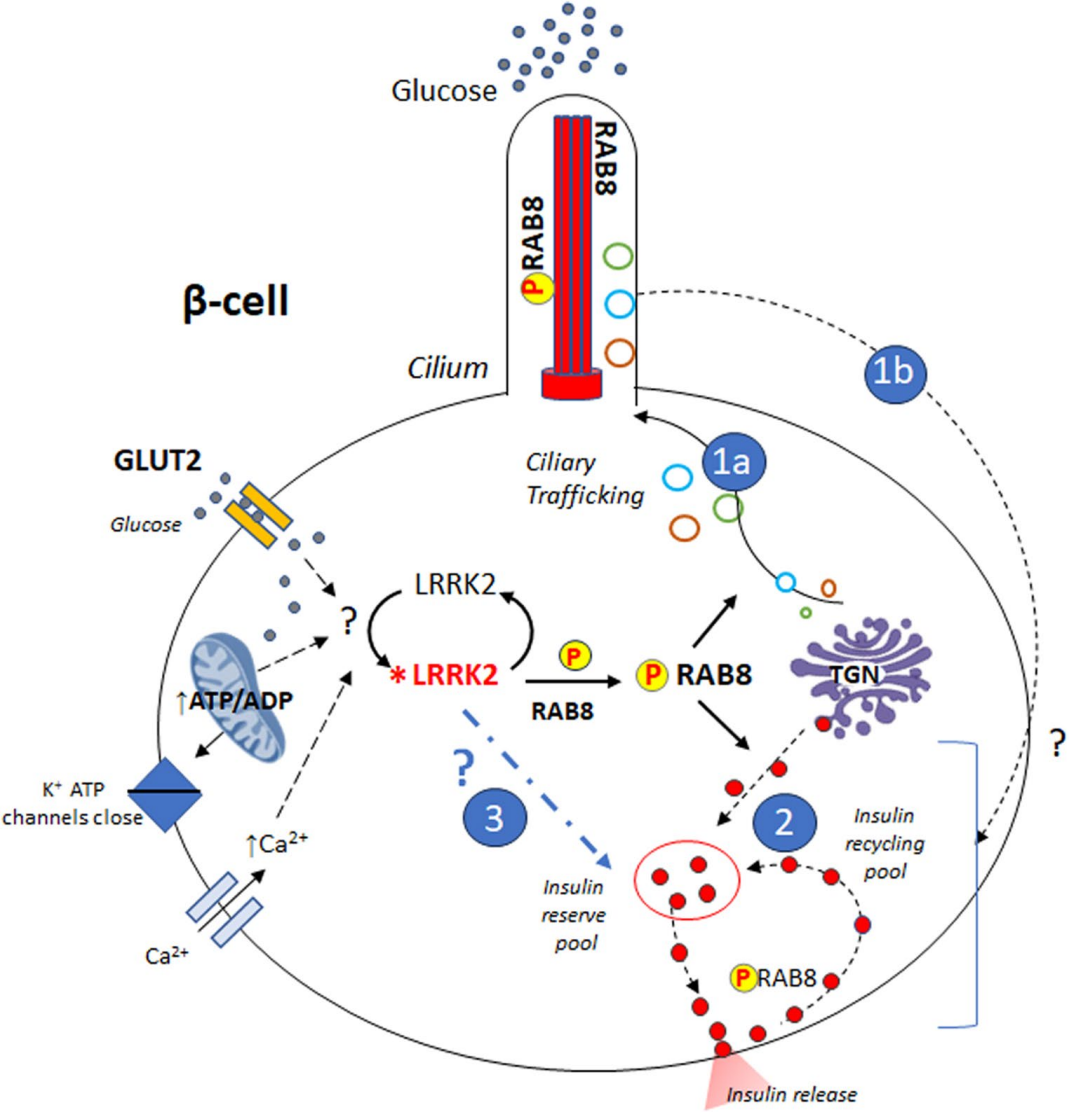
Molecular model of LRRK2 action on insulin secretion in pancreatic β-cells. The data indicate that the LRRK2 kinase, following exposure to high glucose (stimulated condition), phosphorylates RAB8 at threonine 72. Once phosphorylated, RAB8 (P-RAB8) promotes insulin secretion via two possible, non-mutually exclusive mechanisms. (1) Indirect mechanism mediated by the primary cilium. Phospho-RAB8 would control the trafficking towards the cilium of vesicles containing proteins that are critical for its formation and function (1a), thereby promoting the regulated insulin secretion by a mechanism that is still poorly understood (1b). (2) Direct mechanism, mediated by the action of phospho-RAB8 on insulin granules. In this case, P-RAB8 could directly promote the trafficking of insulin granules from the TGN (or recycling pool) to the plasma membrane and the hormone secretion. (3) Although not addressed in the study, activation of LRRK2 may promotes insulin secretion also through RAB8 independent pathways. *LRRK2 indicates active LRRK2. Dotted lines indicate not yet identified pathways

In conclusion, we propose that LRRK2 kinase modulates GSIS by controlling the pool of insulin granules available for release through the glucose-dependent phosphorylation of RAB8 and the modulation of ciliogenesis (Fig. 6). This is not the only mechanism by which LRRK2 can promote insulin secretion. Indeed, the expression of RAB8T72A only partially inhibits GSIS and does not abolish ciliogenesis. Clearly, LRRK2 may phosphorylate other RAB and non-RAB proteins involved in insulin secretion. Further work is needed to determine how LRRK2 kinase activity is engaged by glucose stimulation, the role of RAB8 and possibly other LRRK2 substrates in ciliogenesis and to understand how the primary cilium can control stimulated insulin secretion.

### LRRK2 and Insulin dysregulation: implication for Parkinson disease

Our analyses on the mouse model expressing the BAC human LRRK2 G2019S mutant confirm the relevance of LRRK2 on pancreatic β-cell function *in vivo*. The LRRK2 G2019S mice show an overt metabolic defect, characterised by mild hypoglycaemia and hyperinsulinemia, a phenotype not previously observed in other LRRK2-related PD models. Modelling LRRK2 PD has been proven challenging [65]. However, the BAC model used in this paper had shown bona fide PD phenotypes, including motor impairment and nigral damages [31, 37] prompting further studies analysing the impact of LRRK2 on glucose homeostasis.

The BAC strategy implies the expression of the human mutant protein on the top of endogenously expressed rodent LRRK2, therefore the different amount of protein together with the altered kinase activity may account for the metabolic phenotype observed specifically in our model. In line with this, a recent study correlates increased LRRK2 protein expression and obesity in mice [66].

Our data suggest that the metabolic alterations observed in our model result from a pancreas-specific mechanism, primarily a dysregulated insulin release, rather than to an indirect, nervous system-mediated effect.

How does altered insulin release relate to Parkinson’s disease pathology? Cumulating evidence indicates a direct link between altered insulin signalling and neurodegeneration [67]. In the CNS, insulin is essential for neuronal survival and function, regulating synapse formation and plasticity, neuroinflammation and oxidative stress [68–70]. It acts via specific receptor systems that are abundant in the basal ganglia and *substantia nigra*, the brain areas most affected by PD. Reduced expression of insulin receptor mRNAs [71] and decreased insulin action (insulin resistance) have been documented in the CNS of PD patients [72, 73] and there is evidence that this has a direct impact on dopaminergic function, accelerating the progressive neuronal degeneration and the development of PD deficits [74].

Our results posit the PD-related protein LRRK2 at the center of this potential pathological mechanism. We provide evidence that LRRK2 kinase activity affects not only CNS functions but also insulin release and glucose homeostasis. Indeed, mice carrying the PD-linked G2019S mutation, characterized by increased kinase activity, demonstrate higher insulin blood levels. Insulin hypersecretion might lead to insulin receptors downregulation as an adaptive response to prevent metabolic stress. All tissues expressing insulin receptor pathways would be affected, including the brain, thus accelerating the neurodegenerative process. This process has been clearly observed in studies that artificially increased insulin in the circulation in rodents and men [75, 76]. Consistent with this possibility, our preliminary data indicate that LRRK2 G2019S mice exhibit islet hyperplasia as early as three months of age, before the nigrostriatal distress and onset of motor defects observed at six months [37]. Longitudinal studies monitoring β-cell activity, insulin resistance and motor behaviour will be essential to establish causality among these events and hence optimize preventive treatments for PD.

Supporting our hypothesis are data derived from LRRK2 variant carriers, who have a higher prevalence of prediabetes and elevated triglyceride levels compared to non-carriers and individuals with PD due to other genetic mutations [77]. A recent comprehensive lipidomic study of serum and cerebrospinal fluid from large, multicentre cohorts of PD patients (and matched controls) with and without the LRRK2 G2019S mutation has revealed that LRRK2-PD patients have unique lipid profiles differing from those of idiopathic PD patients and healthy controls. These profiles can distinguish asymptomatic LRRK2 carriers from non-carriers, suggesting that metabolic changes may occur even before the onset of motor symptoms [78, 79]. Notably, pathway analysis of the altered lipid species found in LRRK2 G2019S mutation carriers and non-carriers revealed that lipids involved in insulin and glucose pathways significantly discriminate between the two groups [78]. Furthermore, a significant interaction between the LRRK2 SNP rs10506151 and T2D has been demonstrated [80].

In conclusion, this study presents a new pathway regulating insulin secretion, involving LRRK2, RAB8, and ciliogenesis. Our findings suggest that LRRK2 might be directly involved in the abnormalities of insulin signaling observed in PD patients. The therapeutic effect of drugs targeting insulin resistance in PD management reinforces this association [81–84]. Both T2DM and PD are common disorders that negatively impact patients’ quality of life. It is therefore extremely essential to investigate these diseases considering their associations and interactions.

## Electronic supplementary material

Below is the link to the electronic supplementary material.

Supplementary Material 1

Supplementary Material 2

## Data Availability

The datasets generated and analysed in the current study are available from the corresponding author on reasonable request.

## Abbreviations

CNS: central nervous system
GDI: GDP dissociation inhibitors
GFP: green fluorescent protein
GSIS: glucose-stimulated insulin secretion
HOMA-β: homeostasis model assessment of β-cell function
HOMA-IR: Homeostasis Model Assessment of Insulin Resistance
LRRK2: Leucine-rich repeat kinase 2
PD: Parkinson’s disease
RIPL1/2: Rab Interacting Lysosomal Like Protein 1/2
SNARE: Soluble N-ethylmaleimide-sensitive factor attachment protein receptors
T2DM: type 2 diabetes mellitus
TBS: Tris-buffered saline
TGN: trans Golgi network
TIRF: total internal reflection fluorescence
