# Supplementary figures and images for: LRRK2 kinase modulates glucose-stimulated insulin secretion via RAB8 phosphorylation and ciliogenesis
Source: Cell Mol Life Sci. 2025 Jul 17;82(1):276. doi: 10.1007/s00018-025-05810-w (PMC12270992; doi:10.1007/s00018-025-05810-w)

Figure 2B and 2D

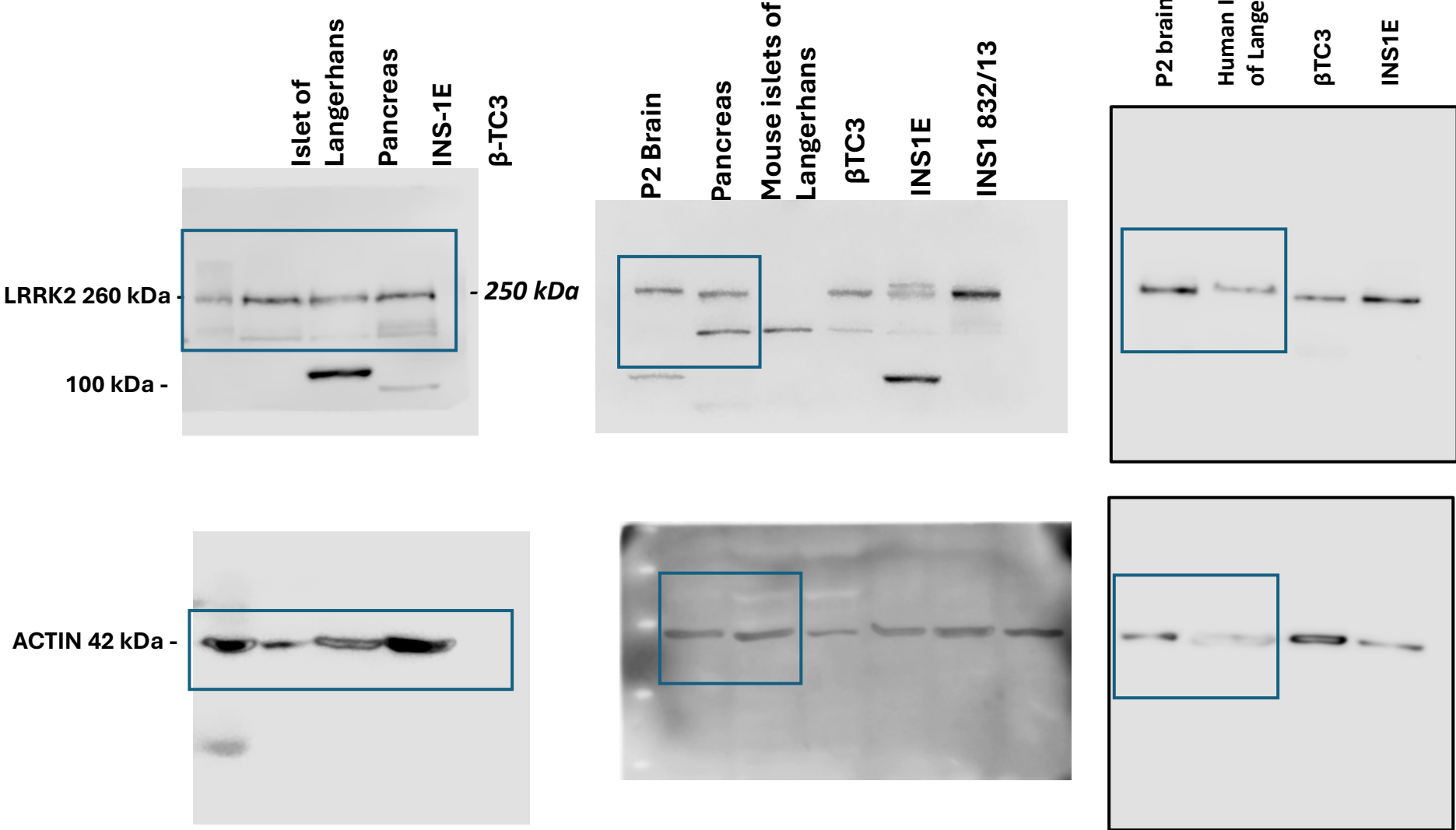

Figure 4A

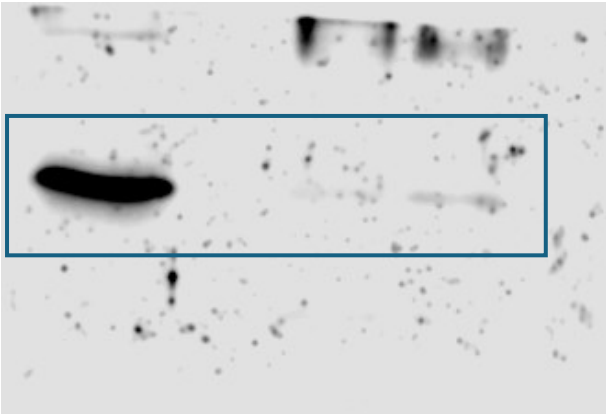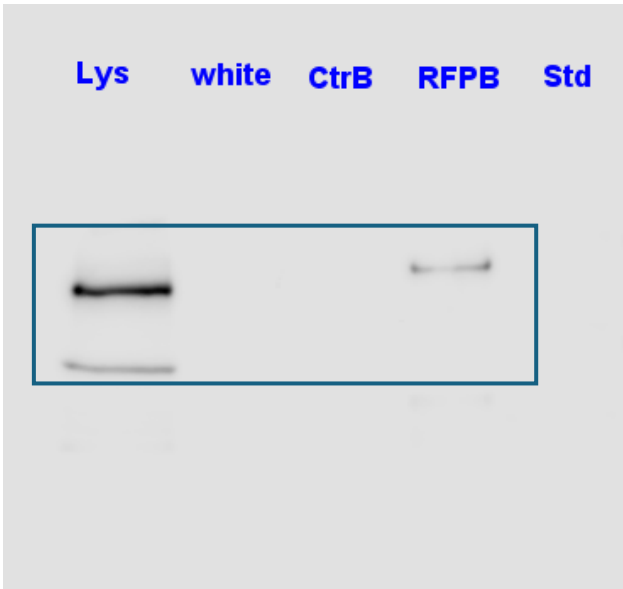

Figure 4B

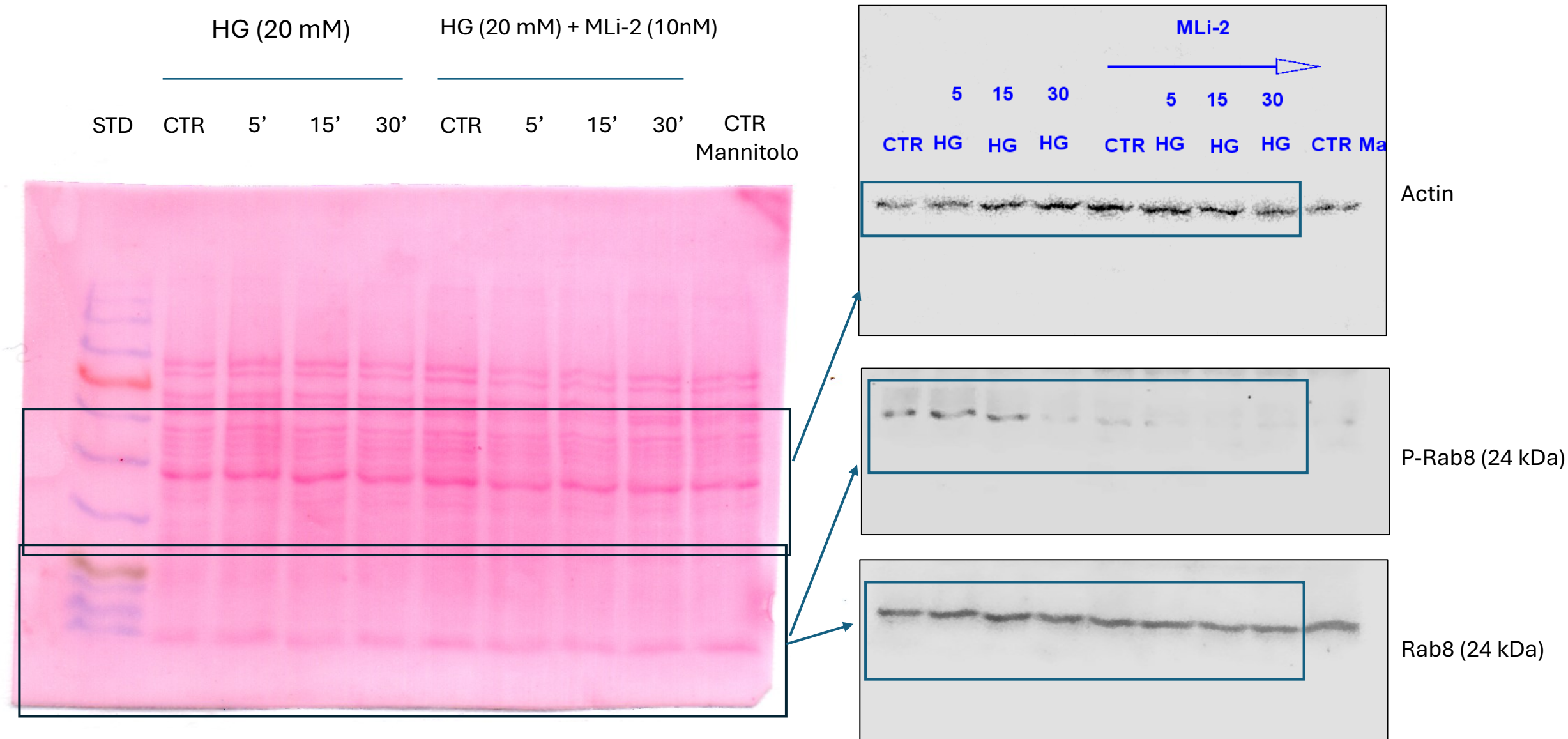

Figure S5A

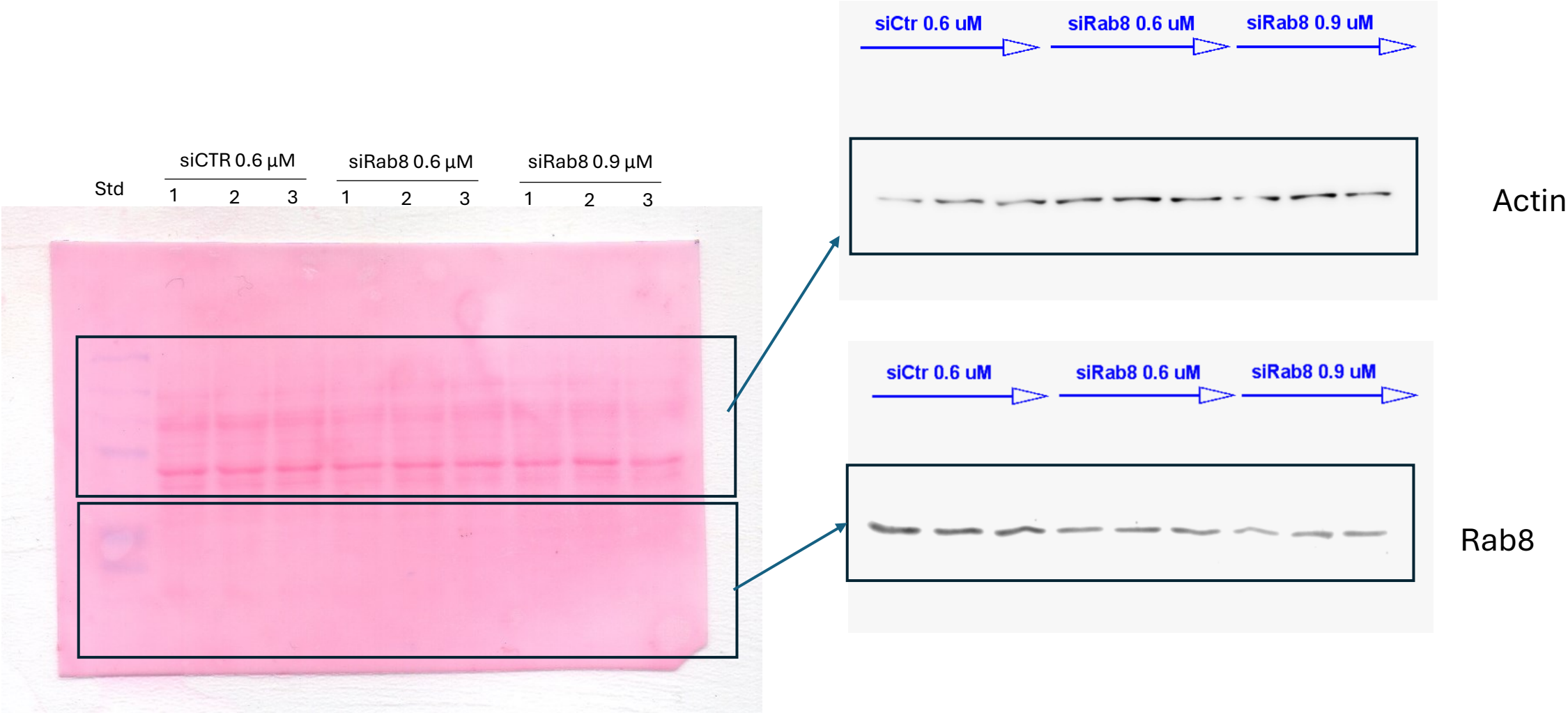

Supplement: Supplementary file 1 — Supplementary Material 1 [file 18_2025_5810_MOESM1_ESM.pdf]
