# Supplementary material for: LRRK2 kinase modulates glucose-stimulated insulin secretion via RAB8 phosphorylation and ciliogenesis
Source: Cell Mol Life Sci. 2025 Jul 17;82(1):276. doi: 10.1007/s00018-025-05810-w (PMC12270992; doi:10.1007/s00018-025-05810-w)
Supplement: Supplementary file 2 — Supplementary Material 2 [file 18_2025_5810_MOESM2_ESM.pdf]

LRRK2 kinase modulates glucose-stimulated insulin secretion via RAB8 phosphorylation and ciliogenesis

Dule Nevia[1]*, Marku Algerta[1]*, Galli Alessandra[1], Pischedda Francesca[2], Adriano Lama[2], Castagna Michela[1], Marciani Paola[1], Bertuzzi Federico[3], Piccoli Giovanni[2] and Perego Carla[1]#

[1] Laboratory of Molecular and Cellular Physiology, Department of Excellence of Pharmacological and Biomolecular Sciences, Università degli Studi di Milano, Milan, Italy
[2]CIBIO, Università Degli Studi di Trento, Italy & Dulbecco Telethon Institute, Trento, Italy
[3]Niguarda Cà Granda Hospital, Milan, Italy

Supplementary Figures

Figure S1

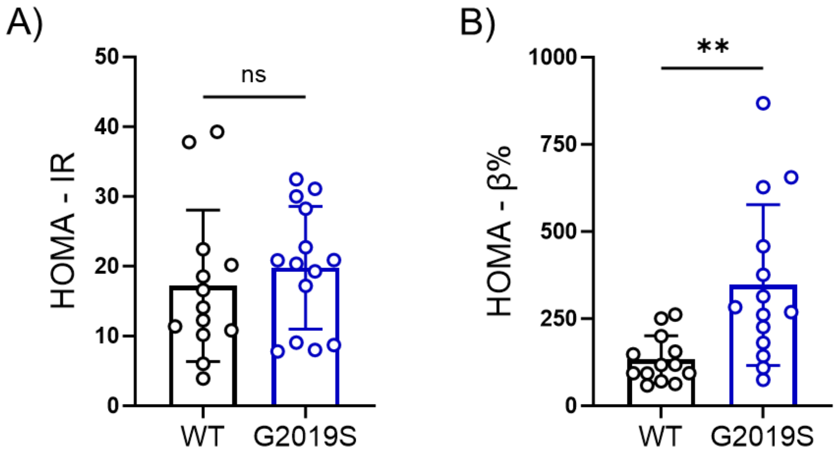

Fig. S1 Quantitative analysis of HOMA IR (**A**) and HOMA-β (**B**) (n = 13 WT and 14 G2019S mice). Blood glucose levels were measured under fasting conditions (6 hrs, light cycle). HOMA were computed as follow: HOMA-IR = [fasting insulin (μU/mL) × fasting glucose (mmol/L)]/22.5; HOMA-β = [20 × fasting insulin (μU/mL)]/[fasting glucose (mmol/L)-3.5]. Data are reported as mean ± SD. Student's two tailed T-test: **p<0.01; ns: not significant.

Figure S2

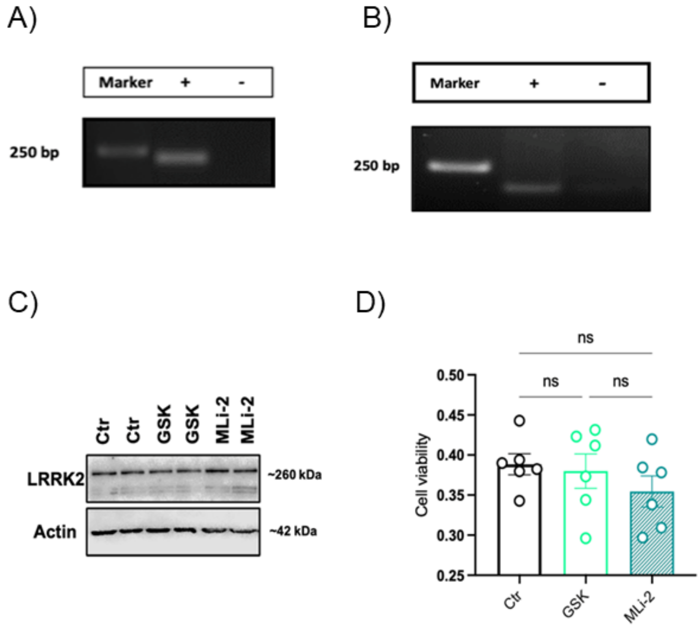

Fig. S2 LRRK2 expression. **A** Evaluation of LRRK2 expression in βtc3 cells by means of RT-PCR. **B** Evaluation of LRRK2 expression in isolated human islets of Langerhans by means of RT-PCR. **C** Western blot analysis of LRRK2 expression in βtc3 cells incubated in the presence or absence of LRRK2 kinase inhibitors GSK (200 nM) and MLi-2 (10 nM). Actin was used as loading control. **D** βTC3 cells were treated for 45 minutes with LRRK2 inhibitors and cell viability was assessed by incubating the cells with 0.5 mg/mL MTT (3-(4,5-dimethyltiazol-2-yl)-2,5-diphenyltetrazolium bromide) (M5655, Sigma-Aldrich) for 4 hours at 37 °C in a 5% CO_2 incubator (n = 6 independent experiments). Data are reported as mean ± SD. ns: not significant.

Figure S3

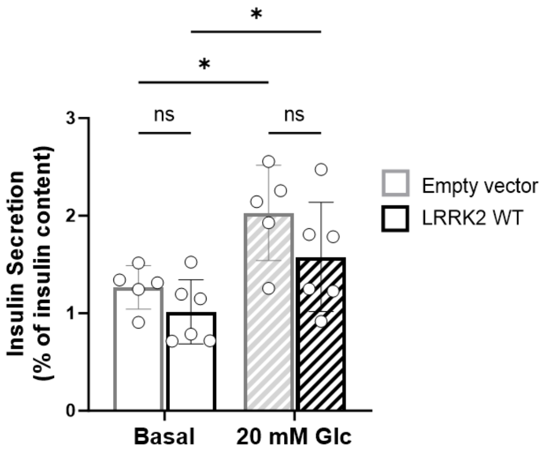

Fig. S3 LRRK2 WT overexpression does not modify the insulin secretion. Glucose-stimulated (20 mM) insulin secretion in βtc3 cells transfected with LRRK2 WT or an empty vector. Data are expressed as a percentage of insulin content and are reported as mean ± SD. (n = at least 5 independent experiments). Two-way ANOVA: *$p<0.05$; ns: not significant.

Figure S4

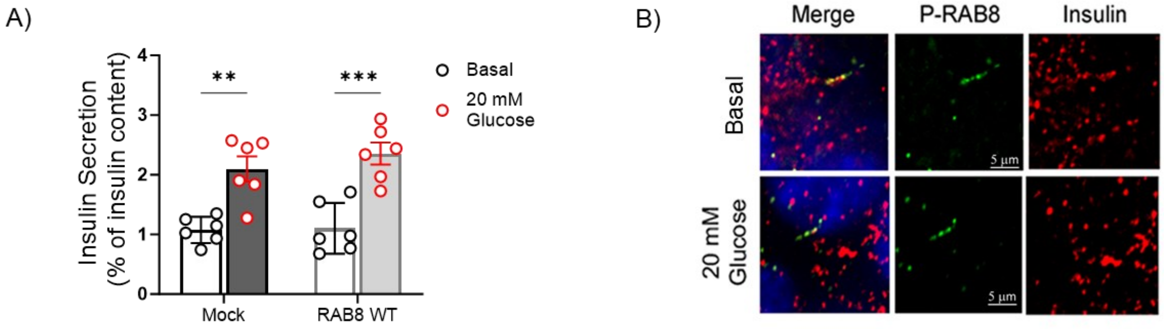

Fig. S4 RAB8 and insulin. **A** Glucose-stimulated (20 mM) insulin secretion in naive βtc3 cells (Mock) or βtc3 cells transfected with RAB8 WT. Data are expressed as percentage of insulin content (*n* = 6 independent experiments) and are reported as mean ± SD. Two-way ANOVA: **p<0.01; ***p < 0.005. **B** Representative immunofluorescence images of βtc3 cells stained with DAPI (blue), anti-pThr72-RAB8 (P-RAB8 - green) and anti-insulin (red) antibodies under basal (1 mM glucose) and stimulated (20 mM glucose) conditions. Scale bar: 5 µm.

Figure S5

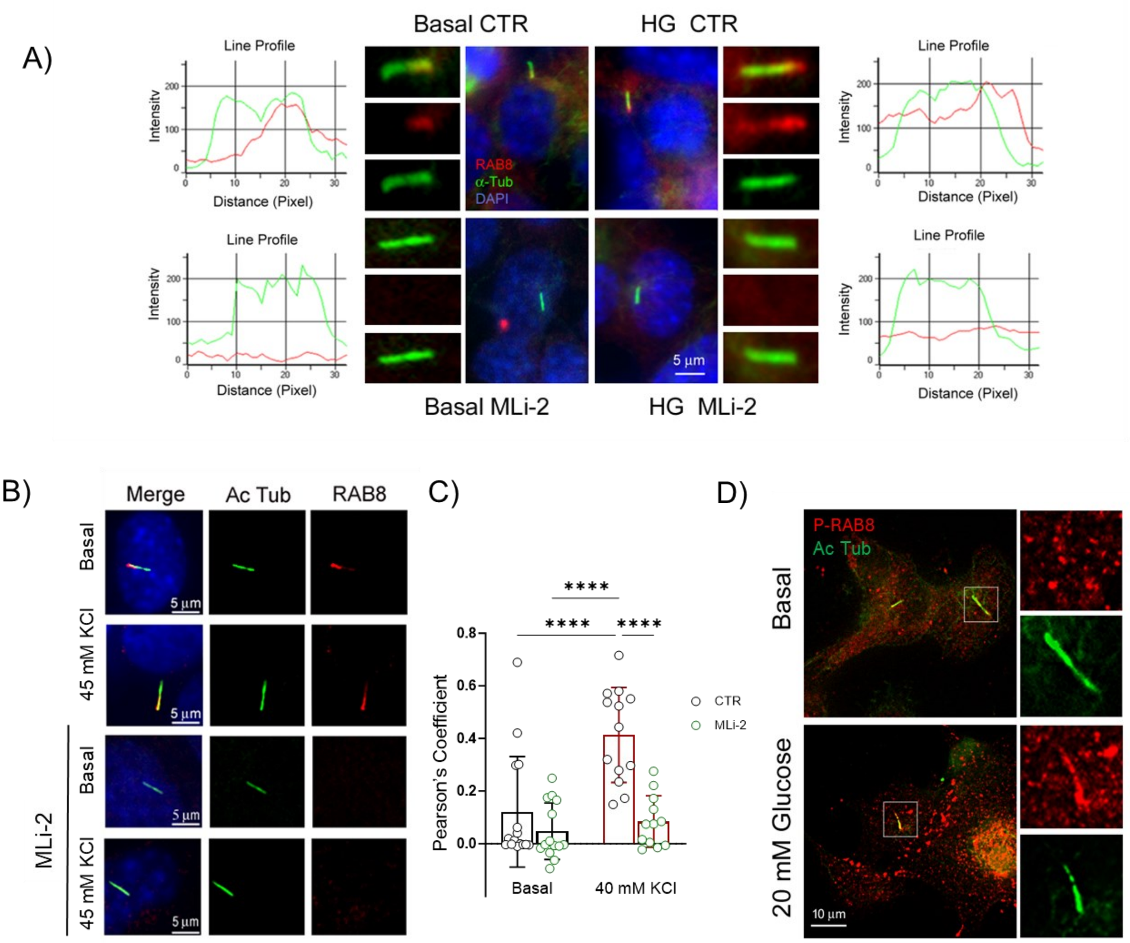

Fig S5. RAB8 localization under stimulated conditions. **A** Representative triple immunofluorescence images of βtc3 cells stained with DAPI (blue), anti-acetylated tubulin (green) and anti-RAB8 (red) antibodies. The cells were maintained under basal (1 mM glucose) or hyperglycaemic (HG - 20 mM glucose) conditions in the presence/absence of MLi-2 (10 nM) for 60 minutes. Scale bar: 5 µm. A higher magnification view of the cilium (3x) is shown. The red and green line profiles of stainings in the ciliary axis are shown in the figure. **B** Representative triple immunofluorescence images of βtc3 cells stained with DAPI (blue), anti-acetylated tubulin (green) and anti-RAB8 (red) antibodies. Cells were incubated in basal (1 mM glucose) or stimulated (40 mM KCl) conditions in the presence/absence of MLi-2 (10 nM) for 60 minutes. Scale bar: 5 µm. **C** Colocalization between acetylated tubulin and RAB8 following KCl stimulation was quantified by means of the Pearson's coefficient analysis (n = at least 13 cells per condition). Data are expressed as mean ± SD. Two-way ANOVA: ****$p < 0.001$. **D** Representative immunofluorescence images of βtc3 cells double stained with anti-acetylated α-tubulin (green) and anti-pThr72-RAB8 (red) antibodies. βtc3 cells under basal (1 mM glucose) and stimulated (20 mM glucose) conditions. The colocalization between acetylated tubulin and RAB8 is shown in yellow/orange. Scale bar: 10 µm**.** A particular of the image is shown at higher magnification (3x).

Figure S6

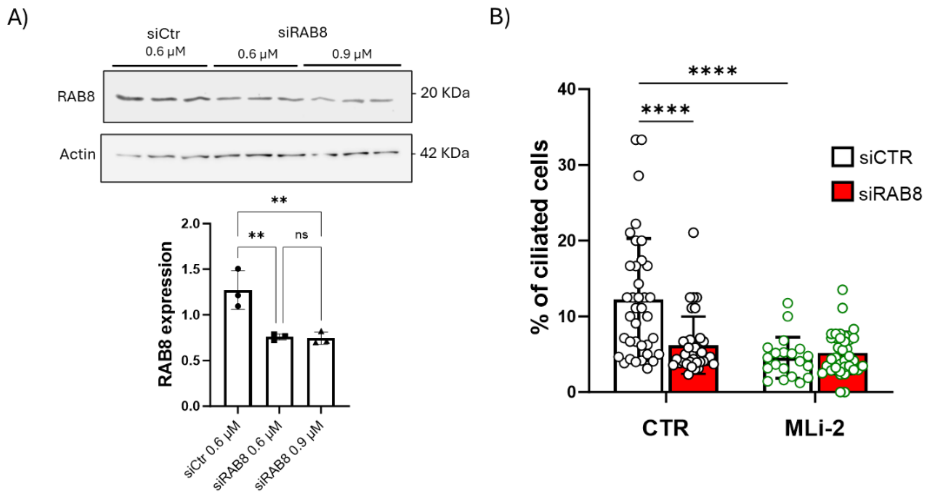

Fig S6. A RAB8 and ciliogenesis. βTC3 cells were transfected with 0.6 μM Silencer™ Negative control (siCtr), 0.6 and 0.9 μM RAB8 siRNA (siRAB8) and 48 hrs after transfection cells were lysed and analyzed. Representative immunoblots of RAB8 and relative quantification of bands. Actin was used as loading control. Two-way ANOVA, **p<0.01. **B** Quantification of ciliated β-TC3 cells after RAB8 silencing using a siRNA strategy, in serum-fed conditions plus 1 mM glucose, in the presence or absence of MLi-2 (10 nM; 60-minute treatment) (n = at least 37 image fields per condition). Data are reported as mean ± SD. Two-way ANOVA, ****p < 0.001.
